# Subgroup identification using individual participant data from multiple trials: An application in low back pain

**DOI:** 10.1017/rsm.2025.10010

**Published:** 2025-06-18

**Authors:** Cynthia Huber, Tim Friede

**Affiliations:** Department of Medical Statistics, University Medical Center Göttingen, Göttingen, Lower Saxony, Germany

**Keywords:** GLMM-tree, heterogeneity, individual-participant data (IPD), meta-analysis, metaMOB, model-based recursive partitioning (MOB), subgroup identification

## Abstract

Model-based recursive partitioning (MOB) and its extension, metaMOB, are tools for identifying subgroups with differential treatment effects. When pooling data from various trials the metaMOB approach uses random effects to model the heterogeneity of treatment effects. In situations where interventions offer only small overall benefits and require extensive, costly trials with a large participant enrollment, leveraging individual-participant data (IPD) from multiple trials can help identify individuals who are most likely to benefit from the intervention. We explore the application of MOB and metaMOB in the context of non-specific low back pain treatment, using synthetic data based on a subset of the individual participant data meta-analysis by Patel et al. ^1^ Our study underscores the need to explore heterogeneity in intercepts and treatment effects to identify subgroups with differential treatment effects in IPD meta-analyses.

## Highlights

### What is already known?

- The identification of subgroups of individuals benefitting in particular from an experimental intervention is of interest in many fields. However, a single trial is often too small for this purpose implying the use of individual participant data (IPD) from multiple trials.
- Ignoring the between-trial heterogeneity in subgroup identification may lead to spurious subgroup findings. metaMOB was developed to overcome this problem.
- metaMOB extended model-based recursive partitioning to IPD meta-analysis.

### What is new?

- We present an application of metaMOB identifying subgroups in IPD from four randomized controlled trials in chronic low back pain.
- Results previously known from simulations only were now demonstrated on real-world data.

### Potential impact for RSM readers

- Readers will understand how to approach the identification of subgroups using data from multiple trials with the metaMOB method; the code for reproducing the illustrated example is available in the Supplementary Material.

## 1 Introduction

For interventions with small to moderate treatment benefits, investigators often aim to identify particular patient subgroups that could potentially derive a greater treatment advantage.^2^ If in addition to the smaller overall treatment benefit the trial sizes are small due to time and cost reasons, pooling data from several trials for the analyses is attractive. Different methods have been proposed for this purpose; see, for example, Wang et al.,^3^ ^,^ ^4^ Mistry et al.,^5^ and Huber et al.^6^

For non-specific low back pain (NSLBP), a repository of individual participant data from 19 completed NSLBP trials testing similar nonpharmacological interventions was set up to investigate which patients are more likely to benefit from treatment in terms of the back-related disability.^1^

For subgroup identification, Seibold et al.^7^ investigated the use of model-based recursive partitioning (MOB), a versatile tree-based method combining parametric models with recursive partitioning.^8^ MOB showed an overall good performance in neutral comparison studies^9^ ^–^ ^12^ assessing the performance of subgroup identification approaches in precision medicine. With several trials rather than a single trial, trial effects would need to be accounted for by stratification or study-specific intercepts; these could be included in MOB. However, MOB in its original form cannot account for between-study heterogeneity in regression coefficients. For the identification of subgroups based on individual participant data from multiple studies metaMOB, a generalized linear mixed model tree approach,^13^ was investigated by Huber et al.^6^ The metaMOB approach relies on one-stage IPD meta-analysis models with different options to model the baseline heterogeneity as investigated by Legha et al.^14^ Analyzing IPD in meta-analysis, including the use of tree-based methods like metaMOB accounting for two distinct types of heterogeneity, baseline, and treatment effects, is essential in the context of IPD meta-analysis.^6^

In this article, we illustrate the use of different MOB approaches that use different approaches for accounting for the different types of heterogeneity on synthetic low back pain data based on the data set collected by Patel et al.^1^

## 2 Low back pain data

Patel et al.^1^ collected data from 19 completed NSLBP trials to identify participant characteristics predicting clinical response to treatments for low back pain. Following a data-sharing agreements, we obtained data from 8 out of the 19 eligible NSLBP trials. As identifying subgroups with differential treatment effects requires data from two-arm trials, with consistent measurement of outcome variables and subgroup-defining factors across all trials, the number of trials available for the subgroup identification is further reduced. We focused on two-arm trials that included the Roland Morris Disability Questionnaire (RMDQ) measured at baseline, denoted as RMDQ_0, and at least one follow-up visit. Therefore, from the eight eligible NSLBP trials, we selected four trials for our analysis including 1780 individuals. To enable data publishing and reproducibility, we created a synthetic data set of these four trials using the R-package synthpop with the option method="parametric". The synthesis of the data results in randomization ratios that are comparable to those in the underlying real trials, as well as similar distributions for the continuous covariates. However, these ratios and distributions are not perfectly matched. In particular, the randomization ratios may yield values that appear unusual. Nevertheless, this discrepancy does not impact the main objectives of illustrating different subgroup identification approaches. The synthetic data and the R code used for the analyses in the subsequent sections can be found in the Supplementary Material.

Table 1 presents demographics and the RMDQ stratified by trial. The RMDQ score ranges from 0 to 24 with higher values indicating a poorer outcome associated with back pain. The table illustrates that the trials are of different sizes. The largest trial, trial with ID 1, includes more than 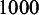 patients, while trial 3 involves only 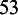 patients. RMDQ was measured at different follow-up visits. Our analyses are based on the data summarized in Table 1. The synthetic data include four studies in which RMDQ is measured at baseline (RMDQ_0) and at least at one follow-up visit. As outcome, we considered the last observation of RMDQ of each patient.

**Table 1:** Summary of the synthesized analysis set

Variable	*N*	Trial 1 (*N* = 1,087)	Trial 2 (*N* = 232)	Trial 3 (*N* = 53)	Trial 4 (*N* = 176)
Categorical variables (*n*, %)
Male	1,548	473 (44%)	131 (56%)	37 (70%)	61 (35%)
Intervention	1,548	805 (74%)	110 (47%)	22 (42%)	82 (47%)
Continuous variables (Median [25%, 75%])					
RMDQ_0	1,537	8.0 [5.0, 12.0]	13.0 [10.0, 17.0]	14.0 [9.0, 16.0]	5.0 [4.0, 8.0]
RMDQ at last follow-up	1,523	4.0 [1.0, 8.0]	1.0 [0.0, 3.0]	5.0 [3.0, 8.0]	2.0 [0.0, 5.0]
Age (years)	1,548	44 [35, 52]	41 [32, 50]	44 [34, 53]	40 [34, 49]

*Note*: Categorical variables are presented as *n* (%), while continuous variables are reported as median [25%, 75% quantile]. Each trial is depicted in a separate column.

Table 1 illustrates that the baseline characteristics, sex, age, and RMDQ at baseline slightly differ between trials. We investigate the treatment effect in the pooled data. Using trial as fixed effect in the linear model as shown in the row “Linear Model Adjusted for Trial,” shows a significant reduction of the RMDQ in the intervention compared to the control arm (see Table 2) indicating a benefit of the intervention over the control treatment. The RMDQ scores at last follow-up differ across trials as indicated by the coefficients of the trial indicators. To account for heterogeneity in the treatment effect using random effects, we applied a linear mixed model to the entire pooled population, incorporating the treatment indicator as random effects and the trial indicator as fixed effect. The variance of the random treatment effect was estimated to be larger than zero, see Table 2. By accounting for between-trial variability in the treatment effect, this model introduces greater uncertainty in the pooled estimate of the treatment, resulting in a wider confidence interval compared to the model presented in the first row of Table 2.

**Table 2:** Treatment effect estimated by an unadjusted linear model, a linear model including the trial indicator as fixed effect and a linear mixed model with treatment as random and trial indicator as fixed effect

Model name	Characteristic	Coefficient	95% CI	*p*-Value
Linear model adjusted for trial	Intervention	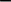 1.1	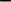 1.6, 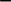 0.64	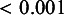
	Baseline RMDQ	0.40	0.35, 0.46	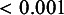
	Trial indicator 2	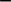 5.8	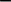 6.5, 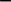 5.1	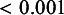
	Trial indicator 3	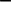 2.2	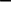 3.6, 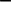 0.87	0.001
	Trial indicator 4	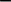 1.3	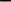 2.1, 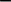 0.56	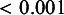
Linear mixed model	Intervention	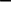 1.1	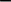 3.0, 0.82	0.12
	Baseline RMDQ	0.40	0.35, 0.46	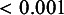
	Trial indicator 2	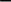 6.4	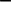 13, 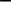 0.22	0.048
	Trial indicator 3	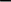 2.4	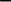 7.3, 2.4	0.15
	Trial indicator 4	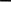 1.5	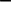 9.3, 6.3	0.3
	Variance for intervention	0.137		

## 3 Subgroup identification

We illustrate the identification of subgroups in NSLBP with differential treatment effects using MOB approaches. MOB and its extensions, as metaMOB or palmtree,^15^ are based on generalized linear models. To describe the different variations of MOB and the underlying models, we assume that the outcome RMDQ is denoted by *y*. Furthermore, the treatment indicator is denoted by *t*, the covariates age, sex, and RMDQ at baseline are denoted by 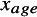 , 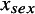 , and 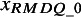 . The baseline covariates 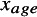 , 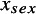 , and 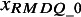 are considered as potential splitting variables and are therefore not involved in the underlying regression model. The analysis includes four trials 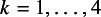 . The corresponding trial indicator is denoted by 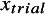 .

For MOB, the outcome of each subgroup *j* and trial *k* is modeled by 
(1)

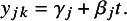

MOB does not account for the data being pooled from different trials; therefore, the right-hand side of the equation does not depend on *k*. Adjusting for the different trials by fixed effects using MOB is referred to as MOB-SI. SI refers to a stratified intercept as for each trial a separate intercept is estimated. The linear model for RMDQ fitted in each subgroup *j* based on the method MOB-SI is 
(2)

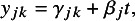

with 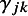 describing the subgroup and trial-specific fixed intercept. Both MOB and MOB-SI can be fitted using the lmtree function of the partykit package. Addressing heterogeneity in the baseline with random intercepts can be achieved by applying MOB-RI. MOB-RI is based on GLMM-trees. Therefore, for analyzing the NSLBP data with RMDQ as outcome, a linear mixed model is fitted to each subgroup *j* and trial *k*: 
(3)

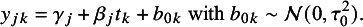



The random intercept 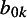 is considered to be the same for each subgroup. Accounting additionally for heterogeneity in the treatment effect is feasible using the following extended metaMOB approaches for IPD meta-analyses, namely metaMOB-RI and metaMOB-SI.

metaMOB-RI: 
(4)

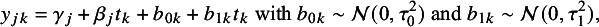

and metaMOB-SI: 
(5)

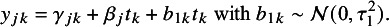

The approaches involving random effects, MOB-RI, metaMOB-SI, and metaMOB-RI are fitted using the lmertree function of the glmertree package.

An alternative to both the MOB-RI and MOB-SI approaches, which address heterogeneity in baseline by using subgroup and trial-specific intercepts (MOB-SI) or random intercepts for the trial indicator (MOB-RI), is the Generalized Linear Model Trees with global additive effects, referred to as *palmtree*.^15^ In contrast to MOB-RI which assumes the random intercepts to be constant across subgroups, *palmtree* includes the treatment indicator in the model similar to MOB-SI, but assumes these intercepts to be the same across the identified subgroups: 
(6)

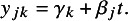



The regression models used for the metaMOB approach (Equations (4) and (5)) are in alignment with the models typically used in random-effects meta-analysis, here the normal–normal hierarchical model.

### 3.1 MOB

**Figure 1:**
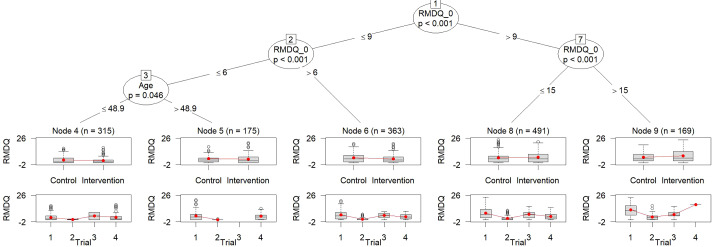
Tree obtained by MOB-SI. *Note*: Five subgroups are identified. The upper boxplots display the outcome values stratified by the treatment indicator, while the lower boxplots show the outcome values stratified by the trial indicator. Note that the RMDQ, illustrated on the *y*-axis, ranges from 0 to 24, with higher values indicating a poorer outcome. To the best of our knowledge, the *y*-axis limits are hard-coded and cannot be manually adjusted.

For the NSLBP data, approaches that account for between-trial heterogeneity are more suitable due to various differences between the trials included. Therefore, MOB using Equation (1) is not employed for data analysis. MOB-SI accounts for heterogeneity in the baseline by adjusting for the trial indicator, see Equation (2). The result of this approach is illustrated in Figure 1. The subgroups are defined by the RMDQ_0 and Age. MOB-SI partitions the group of participants with RMDQ_0 values larger than 9 into three subgroups by additional splits on RMDQ_0 and Age. MOB-SI estimates the treatment effect separately in each of the identified subgroups using a linear model with treatment and trial as factors (see Equation (2)), resulting in *p*-values for the treatment effect within two subgroups, denoted node 4 and node 6, that are smaller than 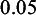 . Both of these subgroups estimate a reduction of the RMDQ score of the intervention compared to the control indicating a treatment benefit. In node 6, which includes only subjects with baseline RMDQ values greater than 6 and less than or equal to 9, the estimated benefit of the intervention is the largest, with a predicted reduction of 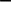 1.66 points in RMDQ in the intervention group compared to the control group. The estimated treatment effect is based on Equation (2) that is also used for the MOB-SI approach.

**Figure 2:**
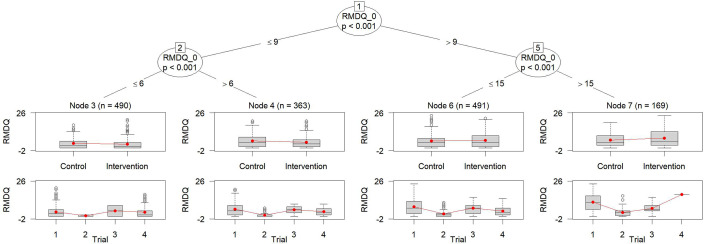
Tree obtained by metaMOB-SI. *Note*: Four subgroups are defined. All splits are performed on the variable RMDQ_0. The upper boxplots display the outcome values stratified by the treatment indicator, while the lower boxplots show the outcome values stratified by the trial indicator. Note that the RMDQ, illustrated on the *y*-axis, ranges from 0 to 24, with higher values indicating a poorer outcome. To the best of our knowledge, the *y*-axis limits are hard-coded and cannot be manually adjusted.

An alternative to MOB with stratified intercept for the trial is the GLMM-tree algorithm with a random intercept to account for baseline heterogeneity. This approach is referred to by MOB-RI,^6^ which imposes a restriction on the heterogeneity of the baseline by assuming a certain distribution of its random effects, see Equation (3). Nevertheless, the subgroups identified by MOB-RI are the same as for MOB-SI. However, the estimated effects of MOB-RI differ slightly from MOB-SI as the model used for the estimation of the treatment effect is based on Equation (3).

### 3.2 metaMOB

Heterogeneity in the treatment was not assumed by the models MOB-RI and MOB-SI. The estimated tree of metaMOB-RI is identical to the ones estimated by MOB-SI and MOB-RI (see Figure 1). The tree estimated by *palmtree* is also consistent with those estimated by MOB-SI, MOB-RI, and metaMOB-RI. When fitting the model that incorporates heterogeneity in both baseline and treatment effects by employing random effects for both, the estimated variance of the random treatment effect is equal to zero. Although the identified subgroups are consistent across these approaches, the estimated effects differ slightly. This is because each MOB approach uses a different model to estimate the effects.

Huber et al.^6^ recommended to use metaMOB-SI as it is the most flexible approach and showed the best performance regarding different measures, for example, false discovery rate across different scenarios. Applying metaMOB-SI to NSLBP identifies four subgroups, only. Therefore, it differs from the results obtained by MOB-SI, and metaMOB-RI. The tree obtained by metaMOB-SI is illustrated in Figure 2. The estimated treatment effect in metaMOB-SI underlying linear mixed model is not significant for any of the identified subgroups. In node 4 (as denoted in the figure) the largest treatment benefit is observed. This is consistent with the result obtained by the other MOB approaches as the definition of node 4 of metaMOB-SI, is identical with the definition of node 6 of MOB-SI, see Figures 1 and 2, respectively. The estimated treatment effect for node 4 based on Equation (5) is 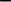 1.45 (*p*-value: 0.07), indicating a reduction of 1.45 points in the RMDQ score for subjects in the intervention arm compared to the control arm, suggesting less pain-related disability or better functional status. The variance of random treatment effect 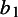 , which is assumed to be constant over the identified subgroups, is estimated to be 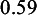 in the underlying mixed model of metaMOB-SI.

## 4 Discussion

When identifying subgroups with differential treatment effects based on data from multiple trials, it is crucial to account for the heterogeneity between these trials.^5^ ^,^ ^6^ ^,^ ^16^ In this manuscript, we illustrated how different modeling approaches for heterogeneity in the intercepts and the treatment effects using the MOB approach can lead to different results based on a subset of synthetic NSLBP data. Due to a limited number of baseline covariates considered to define the subgroups, the results of the different procedures only slightly differ. The approach recommended by Huber et al.,^6^ metaMOB-SI, is the only approach whose results differed from the other approaches accounting for (different types of) heterogeneity. MetaMOB-SI identified fewer subgroups compared to the other approaches which aligns with the results obtained by the simulation study in Huber et al.^6^: Approaches that make assumptions resembling the true underlying heterogeneity structure often result in less complex trees.

Although the metaMOB-SI approach was recommended it also comes with its limitations regarding the estimability of the regression coefficients for the dummy coded trial indicator variable, the stratified intercept. The application of MOB-SI and metaMOB-SI to the NSLBP data encountered the same problem. In node 9 of MOB-SI and node 7 of metaMOB-SI, only a small number of patients from trial 4 were included, not allowing for accurately estimated separate intercepts. The inclusion of only a few observations of a single trial in a node hinders further splitting on these nodes using MOB-SI and metaMOB-SI because the underlying model and, consequently, the splitting criterion cannot be calculated. Although, the choice of whether to model the between-study heterogeneity should ideally be determined a priori, the aforementioned estimation difficulties might require a fallback strategy on a simpler model, that is, a model with less parameters as for instance metaMOB-RI. Nevertheless, metaMOB-SI is recommended as first choice.

The number of trials included in this illustrative analysis is also small. Including a smaller number of trials in a meta-analysis increases the likelihood of heterogeneity estimates for between-trial treatment effects being equal to zero.^17^ Variances estimated of the random treatment effect of zero were observed for the linear mixed model on the overall population, as well as for the model including the identified subgroups of metaMOB-RI. For the mixed model defined in Equation (5) and therefore metaMOB-SI’s underlying model, the random treatment effect variance was not estimated to be equal to zero. Furthermore, some of the methods might not account appropriately for uncertainty in estimating the heterogeneity with only a few studies. Bayesian approaches with weakly informative priors for heterogeneity may offer favorable properties. Extending or modifying metaMOB to incorporate such techniques could be a promising direction for addressing challenges in subgroup identification for IPD meta-analyses with few studies.

For investigating treatment-by-covariate interactions, Riley et al.^18^ recommend, among other considerations, that interaction estimates should be derived solely from within-study information. Estimation and testing methods that adhere to this recommendation are available.^18^ ^,^ ^19^ To the best of our knowledge, no procedures currently exist for subgroup identification in meta-analysis settings using recursive partitioning based solely on within-study information. Incorporating a GLMM that separates within-study and across-study information directly into the metaMOB algorithm is currently not feasible. However, it is possible to estimate treatment effects for the subgroups identified by metaMOB separately from the metaMOB procedure. Specifically, metaMOB can be used solely for subgroup identification, after which GLMMs, as described in Riley et al.^18^ and Godolphin et al.,^19^ can be applied to estimate treatment-by-subgroup effects. Nonetheless, we cannot rule out the possibility that the subgroup identification process in metaMOB is influenced by the amalgamation of within- and across-study information. This issue requires further investigation and may be a topic for future research.

## Supporting information

Huber and Friede supplementary material Huber and Friede supplementary material

## Data Availability

The authors confirm that the data supporting the findings of this study are available within the article and its Supplementary Material.

## Supplementary material

The supplementary material for this article can be found at https://doi.org/10.1017/rsm.2025.10010.
